# Medical and Physical Intelligence

**Published:** 1822-09

**Authors:** 


### MEDICAL AND PHYSICAL INTELLIGENCE.

### 1. *Case of Monstrosity.*

IN May, 1820, a woman of New-York was delivered of a monstrous fœtus, the following description of which is given by Dr. DELAFIELD, of that place. It was formed of two female children joined above the umbilicus, the parts below being perfectly distinct. At first view, it appeared as if there were a single head, attached to two bodies; but, on closer inspection, it was found to be made up of the greater portions of two heads, each looking forward, but in lines which, when produced, would form an acute angle. Posteriorly, two distinct occipita could be perceived, although covered by a common integument, and without any external line of separation between them; while, anteriorly, the faces were so blended as to appear like one. The two mouths were placed together, so as to form a continuous cleft; the two upper lips forming an obtuse angle, and separated by a fissure, extending downwards from the nose. From both mouths probes could be readily passed into the œsophagus, but there the instruments could be felt in contact. The nose also was made up of the larger portions of two, although there was only one complete nostril on each side. On each side of the head was an ear in the usual situation, and at the posterior part of it, at an equal distance from each of these ears, there was another imperfectly formed one, or rather parts of two. Below the neck, a back view of each fœtus was that of a well-formed perfect child, each with its extremities distinct and perfect. Altogether this monster had the appearance of two children placed in contact anteriorly, but the heads turned so as to join at their sides, and all those parts blended together which were in contact.

On removing the integuments of the head, the anterior part appeared as of a single fœtus, the posterior of two. The frontal, temporal, and parietal bones, were formed natural as of one fœtus. The anterior fontanelle was filled by two triangular bones, the line between which was continuous with the sagittal suture. There were two ossa occipitum, well formed and distinct, and between them a large pentagonal bone, about the ordinary size of the parietal. The cerebrum was very large, but appeared only as that of one fœtus; the falx major split into two portions anteriorly, and these portions were attached to the orbitar processes of the os frontis. The tentorium covered two distinct cerebella, from each of which proceeded a medulla oblongata, into the spinal canal of each fœtus. The falx minor made a complete separation between the two cerebella; but there was no process of dura mater between the lobes of either cerebellum.

In the posterior part of the base of the cranium, corresponding to the imperfect ear, which has been noticed, was a bone, into which two auditory nerves passed, and containing a double organ of hearing. It appeared as if made up of the petrous portions of two temporal bones, blended together. It contained two labyrinths, placed in such a manner as that the horizontal canals of the two were in contact, and the cupolæ nearly touched each other. The tympanum, had any existed, would have been between the bones; but this being deficient, the ossicula were also wanting, as well as the foramen ovale, which is usually closed by the base of the stapes. The foramen rotundum did not exist.

On cutting into the chest, two sterna were found, the one anterior, the other posterior, each connecting the ribs of one fœtus to those of the other; the remaining bones of the chest as natural as of two fœtuses. There were two distinct thymus glands, of natural appearance. A process of pleura passed from one spine to the other, as if the two mediastina had united. There were two distinct sets of lungs, each with a distinct trachea, separated by a single œsophagus; but the surfaces of the tracheæ, which are usually anterior, lying upon the spine on each side.

In the anterior of the two large cavities of the chest, formed by the mediastinum, which was described, was a heart of the ordinary size. From it proceeded an aorta, in the usual manner, from the left ventricle, and, after giving off the right subclavian of the right fœtus and both the carotid arteries, it made its curve, and formed the descending aorta of the right child. The artery which went from the right ventricle of this heart, and which is usually the pulmonary, formed an arch, and, after receiving in that situation a considerable branch from the right carotid, and sending off both subclavians of the left child, went to form its descending aorta.

In the other cavity of the chest was another heart, about one-fourth the size of the first, and made up of only one auricle and one ventricle. It gave off two vessels: one of these sent a branch to the aorta of the right fœtus, and, from the point of union, passed off the left subclavian artery of that fœtus; the other vessel going from this heart divided into two branches, which went to form a vertebral artery, going to each foramen magnum.

The larger heart then furnished both aortas, the two carotids distributed as to a single head, both the subclavian arteries of the left child, and the right subclavian of the right child. The smaller heart furnished a single vertebral artery, to enter each foramen magnum, the left subclavian of the right fœtus, and a branch to the aorta.

The vena cava inferior of the left fœtus came up on the left side of the aorta, as high as the usual situation of the left subclavian vein, and, uniting with that vessel, crossed anteriorly to the curve of the aorta, then received the right subclavian of the other child, and turned down to form the superior cava, and enter the right auricle of the larger heart. The vena cava of the right fœtus had the ordinary distribution, and went to form the inferior cava of the larger heart.

The smaller heart received blood only from the veins of the smaller liver.

There was a single umbilicus and cord. The cord was made up of three arteries and one vein; two of the arteries going from the right child, and one from the left. The umbilical vein passed into the liver of the right child; that of the left received no blood from the cord.

From the two conjoined mouths proceeded a common pharynx and œsophagus, which passed down between the tracheæ to a common stomach, of peculiar form. The stomach appeared as if made up of two, united by their smaller curvatures, but with a common cavity; thence proceeded, from the situation where the two pyloric orifices seemed to have joined, a single duodenum. This terminated in an intestine, which was very much convoluted, and, after running about half the length of the small intestines, divided into two portions. After this division, the two intestines terminated each in a distinct colon, which was distributed in the usual manner.

There were two distinct spleens and pancreæ, and each fœtus had also its two kidneys.

There were also two livers, and each of these had its gall-bladder. The liver belonging to the right fœtus was a little larger than usual; its gall-bladder gave off its duct to the duodenum, in the ordinary manner, and contained a little bile. The other liver was about one-fourth the size of the first; its gall-bladder contained no bile, nor was there any duct going from it.

The organs of generation were perfect, and distinct in each fœtus.—
*(American Medical Recorder.)*

### 2. *Medical Virtues of the Apocynum Androsœmifolium.*

The Apocynum Androsœmifolum attracted the attention of Dr. Zollickoffer in the summer of 1817, when, after procuring a suitable quantity of the root, he determined to submit it to a course of experiments, the result of which he noticed in a very succinct manner in a small work, which he wrote and published in the year 1819, entitled "A Materia Medica of the United States, &c." From the attention that Dr. Z. gave to this subject, he perceived that it possessed considerable tonic properties, the action of which depended entirely upon the dose in which it was given; as also that of a valuable emetic, certain, prompt, and effectual in its influence upon the coats of the stomach; rarely occasioning the unpleasant vertigo, and other serious effects, that too frequently accompany the use of some of our indigenous articles belonging to this class of remedies. Dr. Z. gave it in a number of cases of loss of appetite, accompanied with general debility, with decided benefit, in doses of from fifteen grains to twenty, twice and thrice a-day. He is in the habit of exhibiting it in the form of the powdered root, which is to be dried carefully in the shade.

The proper dose in which it should be administered, when given with the view of its producing its effects as an emetic, is from thirty to forty grains at once, or in divided doses of ten grains each, which may be taken every fifteen minutes until it operates. It will be found an invaluable substitute for the pulv. ipecacuanha, when given with a view of exciting diaphoresis, in a combination with opium, in a form somewhat similar to the pulv. dov., which is as follows:

℞ Pulv. Cort. Rad. Apocy. Androsæ, ℨj.

Pulv. Gum. Opii, ℈ss.

Pulv. Nitras Potassæ, ℥ss. M.

In cases of acute rheumatism, when, after venesection has been freely resorted to, with the use of suitable cathartics, a dry surface still continues, this formula, given in doses of ten grains, will seldom fail to induce a general diaphoresis. Dr. Zollickoffer has, in his note-book, two cases of pneumonia, in which venesection had been liberally used, and the pulv. conv. jalap. cum sub. mur. hyd. until the bowels were freely evacuated, without any visible relaxation upon the surface (though with some alleviation of the pain), when it was thought advisable to give ten grain doses every three hours, which ended in a profuse and copious diaphoresis; also one case of phrenitis, in which it was used with the most decided benefit.—*(American Medical Recorder.)*

### 3. *Large Human Calculus.*

A large human calculus has been described by Prof. CUMMING, of Cambridge: it weighs 32 ounces, and measures $15\frac{1}{2}$ inches in circumference. Its specific gravity is 1.756. The nucleus is lithic acid, and to this succeeds a considerable portion of the oxalate of lime, then layers of the triple phosphate, covered by a thick coating of lithic acid, the external surface being composed principally of the fusible calculus. It is in the possession of Trinity College. A calculus is also noticed from the intestines of a hare: it is composed of vegetable matter and the phosphates.—*(Journal of Sciences.)*

### 4. *Size and Shape of the Globules of Blood in different Animals.*

A number of very interesting results have recently been obtained by J. L. PREVOST, M.D. and J. A. DUMAS, respecting the form of the globules of blood of different animals, and the effects of transfusing the blood of one animal into another. The following are their measures of the diameters of the globules:

| | |
|---|---|
| Man, Dog, Rabit, Pig, Hedgehog, Guinea Pig, Muscarden | $\frac{1}{3750}$ of an English inch. |
| Ass | $\frac{1}{4175}$ |
| Cat, Grey Mouse, White Mouse | $\frac{1}{4275}$ |
| Sheep, Horse, Mule, Ox | $\frac{1}{5000}$ |
| Chamois, Stag | $\frac{1}{5450}$ |
| She-Goat | $\frac{1}{7200}$ |

But, while the globules of blood in different animals vary in size, they vary also in form. In the mammalia they are all spherical, while in birds they are elliptical, and vary only in the lengths of their greater axes. They are likewise elliptical in all cold-blooded animals. They found, also, that the colourless globule which exists in the centre of the particles of blood, has the constant diameter of $\frac{1}{7500}$th of an inch in all animals, and whatever be the form of the globule which contains it.

In their experiments on the transfusion of blood, they obtained many interesting results. When animals were bled till they fainted, they died when they were left alone, or when water or serum of blood, at the temperature of 100 Fahr. was injected into their veins. If, on the contrary, the blood of an animal of the same species was injected, every portion of the blood thrown in reanimated the exhausted animal; and, when it had received as much as it lost, it began to breathe freely, to take food, and was finally restored to perfect health. When the injected blood was from an animal of a different species, but whose globules had the same form, though a different size, the animal was only partially relieved, and could seldom be kept alive for more than six days, the animal heat diminishing with remarkable rapidity. When the blood of an animal with spherical globules is injected into a bird, it usually dies under the most violent nervous affections, as if under the influence of the most intense poison; and this takes place even when only a small quantity of blood has been lost. In a great number of cases, cats and rabbits were restored for some days by the injection of the blood of cows and sheep, even when the injection of the blood was not made till twelve or even twenty-four hours after the blood was extracted from the latter. The blood was kept in a fluid state in a cool place, either by taking away a certain quantity of fibrine, or adding 1000th part of caustic soda. When the blood of the sheep was injected into ducks, they died after rapid and strong convulsions.—*(Bibl. Univers.)*

### 5. *Diseases of the Spine.*

We are glad to find that Mr. SHAW has in the press a work on this interesting subject. The first part, we understand, will treat of the Distortions to which young persons are subject in consequence of habitual bad postures and the neglect of proper exercise. Their varieties are to be illustrated by engravings in folio of distorted skeletons, and sketches given of the postures and modes of exercise, and of the application of instruments, calculated to correct each deformity.—The second part will embrace Scrofulous Diseases of the Spine. The extensive collection of specimens of distortion preserved in the Anatomical Museum, Great Windmill-street, give Mr. Shaw peculiar advantages in a work of this kind, and we anticipate a valuable addition to our knowledge on this subject.

### 6. *Ring-Worm.*

In the Monthly Magazine for this month will be found a letter from Mr. T. I. GRAHAM, of Cheltenham, in which he states that he has known the "lime-water procurable from gas-works, and through which the gas has passed for the purpose of purification, perfectly successful in three cases of ring-worm. Two of these were unusually severe, the whole of the scalp being covered with scabs and small deep ulcers. The water has a strong gaseous impregnation, and is most disgustingly fœtid."

### 7. *Mermaid.*

The following extract of a letter from the Rev. Dr. PHILIP, representative of the London Missionary Society at Cape Town, Cape of

Good Hope, dated April 26, 1822, gives a detailed account of a *mermaid*, which was caught in the North of China, and which there is reason to think will be exhibited in London. The proprietor is Captain EADES, of Boston; and the ship which conveys this extraordinary creature to America is to visit the Thames on her way.

"The head is almost the size of that of a baboon. It is thinly covered with black hair, hanging down, and not inclined to frizzle. On the upper lip and on the chin there are a few hairs, resembling those upon the head. The ossa malarum, or cheek-bones, are prominent. The forehead is low; but, except in this particular, the features are much better proportioned, and bear a more decided resemblance to the human countenance, than those of any of the baboon tribes. The head is turned back, and the countenance has an expression of terror, which gives it an appearance of a caricature of the human face; but I am disposed to think that both these circumstances are accidental, and have arisen from the manner in which the creature met its death. It bears the appearance of having died in great agony.

"The ears, nose, lips, chin, breasts and nipples, fingers and nails, resemble those of a human figure.

"The spinous processes of the vertebræ are very prominent, and apparently arranged as in the human body.

"From the position of the arms, and the manner in which they are placed, and from such an examination as could be made in the circumstances in which I was placed at the time I saw it, I can have no doubt that it has clavicles,—an appendage belonging to the human subject, which baboons are without.

"The appearance of the teeth afford sufficient evidence that it is full grown, the incisores being worn on the upper surface. There are eight incisores, four canine, and eight molares. The canine teeth resemble those of a full-grown dog; all the others resemble those of a human subject.

"The length of the animal is three feet; but, not having been well preserved, it has shrunk considerably, and must have been both longer and thicker when alive than it is now. Its resemblance to the human species ceases immediately under the mammæ. On the line of separation, and directly under the breasts, are two fins. From the point where the human figure ceases, which is about twelve inches below the vertex of the head, it resembles a large fish of the salmon species. It is covered with scales all over. On the lower part of the animal the scales resemble those of a fish; but, on that part of the animal which resembles the human form, they are much less, and scarcely perceptible, except on a near inspection. On the lower part of the body it has six fins, one dorsal, two ventrical, two pectoral, and the tail.

"The pectoral fins are very remarkable: they are horizontal, and evidently formed as an apparatus to support the creature when in an erect posture, like that in which it has been sometimes represented combing its hair.

"The figure of the tail is exactly that which is given in the usual representations of the mermaid."

### 8. *Medical Students.*

It is necessary for medical students to be aware that, by the following new regulation of the College of Surgeons, henceforward candidates for the diploma of Surgeon will be prevented from hurrying through all their education in one year, since it obliges them to remain the greater part of two seasons in London. The new regulation will also, we trust, have the effect of inducing them to attend more to medical lectures and medical practice during the summer months.

"From and after the first day of January, 1823, not any candidate for the diploma, who has not regularly attended THREE, or more, Courses of Anatomical Lectures, in London, Dublin, Edinburgh, or Glasgow, will be admitted to examination."

### 9. *Communication between the Auricles of the Heart.*

A preparation, in the possession of FOUQUIER, shows the existence of a free communication between the auricles, without having given rise to blueness of the skin; the patient having laboured under the usual symptoms of aneurism. The same practitioner has likewise shown that the *blue disease* may exist without any such communication. Thus he concludes that, although the organic lesion is often co-existent with this affection, they are not essentially dependant on each other.

### METEOROLOGICAL JOURNAL.

By Messrs. William Harris and Co. 50, Holborn, London.

*From July* 20, 1822, *to August* 19, 1822.

| | Moon. | Rain gauge | Therm. | | | Barom. | | De Luc's Hyg. | | Wind. | | Atmospheric Variation. | | |
|---|---|---|---|---|---|---|---|---|---|---|---|---|---|---|
| | | | 9 A.M. | Max. | Min. | 9 A.M. | 10 P.M. | 9 A.M. | 10 P.M. | 9 A.M. | 10 P.M. | 9 A.M. | 2 P.M. | 10 P.M. |
| July | | | | | | | | | | | | | | |
| 20 | | .03 | 66 | 74 | 60 | 29.42 | 29.43 | 53 | 58 | WSW | W var. | Fine | Sho'ry | Fair |
| 21 | | .09 | 67 | 75 | 59 | 29.45 | 29.50 | 63 | 67 | NW | SW | Fine | Sho'ry | |
| 22 | | .06 | 65 | 68 | 63 | 29.61 | 29.73 | 50 | 52 | WNW | WSW | Showery | Fine | Overc. |
| 23 | | .10 | 65 | 68 | 61 | 29.65 | 29.56 | 56 | 63 | WSW | SW | Cloudy | | Sho'ry |
| 24 | | .02 | 66 | 73 | 61 | 29.53 | 29.50 | 71 | 64 | WSW | W | Showery | Fine | |
| 25 | ☾ | .03 | 64 | 67 | 58 | 29.50 | 29.60 | 67 | 60 | W | W | Showery | Cloud. | Fine |
| 26 | | .05 | 65 | 73 | 58 | 29.62 | 29.61 | 53 | 52 | W | W | Fine | Fair | Cloud. |
| 27 | | .12 | 65 | 73 | 60 | 29.66 | 29.60 | 55 | 59 | W | SSW | Fine | Cloud. | Sho'ry |
| 28 | | .16 | 70 | 68 | 60 | 29.45 | 29.40 | 60 | 60 | W | W | Showery | Rain | Cloud. |
| 29 | | .17 | 64 | 70 | 58 | 29.41 | 29.40 | 63 | 60 | WNW | WNW | Cloudy | Rain | Sho'ry |
| 30 | | .03 | 60 | 64 | 53 | 29.42 | 29.50 | 54 | 58 | WNW | WNW | Cloudy | | Rain |
| 31 | | .19 | 50 | 58 | 48 | 29.54 | 29.56 | 50 | 48 | WNW | W | Fine | Rain | Cloud. |
| Aug | | | | | | | | | | | | | | |
| 1 | | .09 | 56 | 66 | 54 | 29.70 | 29.70 | 63 | 53 | WNW | W | Fine | | Rain |
| 2 | | .06 | 60 | 63 | 49 | 29.60 | 29.77 | 64 | 60 | NW | NE | Cloudy | Rain | Cloud. |
| 3 | ○ | | 60 | 66 | 55 | 29.89 | 29.85 | 63 | 60 | NNW | NNW | Fine | Cloud. | |
| 4 | | | 58 | 68 | 54 | 29.75 | 29.66 | 57 | 46 | E | NE | Fine | | Cloud. |
| 5 | | .05 | 58 | 69 | 56 | 29.72 | 29.82 | 60 | 55 | NE | NNE | Cloudy | | Rain |
| 6 | | | 60 | 68 | 60 | 29.87 | 29.89 | 50 | 45 | N | N | Fine | | |
| 7 | | | 64 | 68 | 55 | 29.94 | 29.92 | 54 | 50 | NW | WNW | Cloudy | | |
| 8 | | | 61 | 71 | 57 | 29.87 | 29.73 | 59 | 58 | SW | ENE | Cloudy | Fine | Fine |
| 9 | | | 63 | 70 | 61 | 29.64 | 29.64 | 55 | 64 | W | WNW | Fine | Cloud. | |
| 10 | ☽ | | 64 | 72 | 63 | 29.66 | 29.70 | 64 | 52 | NW | N | Fine | Fair | Cloud. |
| 11 | | | 65 | 73 | 61 | 29.69 | 29.70 | 50 | 54 | WSW | W | Cloudy | | |
| 12 | | .05 | 63 | 72 | 60 | 29.71 | 29.67 | 60 | 65 | W | W | Fair | | Cloud. |
| 13 | | .03 | 64 | 73 | 62 | 29.66 | 29.81 | 61 | 50 | WNW | W | Showery | Fair | Fine |
| 14 | | | 65 | 72 | 60 | 29.80 | 29.66 | 62 | 60 | WSW | SW | Fine | Fair | Fine |
| 15 | | | 65 | 71 | 55 | 29.77 | 29 82 | 55 | 58 | W var. | WNW | Fair | Fine | Fair |
| 16 | ● | | 60 | 68 | 58 | 29.90 | 29.97 | 45 | 54 | NW | NW | Fine | | |
| 17 | | | 62 | 71 | 61 | 29.90 | 30.01 | 51 | 58 | W | W var. | Fine | | |
| 18 | | | 66 | 75 | 64 | 29.81 | 30.00 | 60 | 61 | W | SSW | Fine | | |
| 19 | | | 67 | 74 | 63 | 30.00 | 30.00 | 65 | 59 | SE | ESE | Fine | | |

The quantity of rain during the month of July, was 2 inches and 75-100ths.

